# MiR‐326/Sp1/KLF3: A novel regulatory axis in lung cancer progression

**DOI:** 10.1111/cpr.12551

**Published:** 2018-11-28

**Authors:** Rong Wang, Jiali Xu, Jing Xu, Wei Zhu, Tianzhu Qiu, Jun Li, Meiling Zhang, Qianqian Wang, Tongpeng Xu, Renhua Guo, Kaihua Lu, Yongmei Yin, Yanhong Gu, Lingjun Zhu, Puwen Huang, Ping Liu, Lianke Liu, Wei De, Yongqian Shu

**Affiliations:** ^1^ Department of Oncology the First Affiliated Hospital of Nanjing Medical University, Jiangsu Province Hospital Nanjing China; ^2^ Department of Oncology Liyang people's Hospital of Jiangsu Province Liyang China; ^3^ Department of Biochemistry and Molecular Biology Nanjing Medical University Nanjing China

**Keywords:** KLF3, lung cancer, microRNA‐326, SP1

## Abstract

**Objectives:**

To investigate the function and regulatory mechanism of Krüppel‐like factor 3 (KLF3) in lung cancer.

**Materials and Methods:**

KLF3 expression was analysed by qRT‐PCR and Western blot assays. The proliferation, migration, invasion, cycle and apoptosis were measured by CCK‐8 and EdU, wound‐healing and Transwell, and flow cytometry assays. The tumour growth was detected by nude mouse tumorigenesis assay. In addition, the interaction between KLF3 and Sp1 was accessed by luciferase reporter, EMSA and ChIP assay. JAK2, STAT3, PI3K and p‐AKT levels were evaluated by Western blot and IHC assays.

**Results:**

The results indicated that KLF3 expression was elevated in lung cancer tissues. Knockdown of KLF3 inhibited lung cancer cell proliferation, migration and invasion, and induced cell cycle arrest and apoptosis. In addition, the downregulation of KLF3 suppressed tumour growth in vivo. KLF3 was transcriptionally activated by Sp1. miR‐326 could bind to 3′UTR of Sp1 but not KLF3 and decreased the accumulation of Sp1, which further indirectly reduced KLF3 expression and inactivated JAK2/STAT3 and PI3K/AKT signaling pathways in vitro and in vivo.

**Conclusions:**

Our data demonstrate that miR‐326/Sp1/KLF3 regulatory axis is involved in the development of lung cancer, which hints the potential target for the further therapeutic strategy against lung cancer.

## 1 INTRODUCTION

Lung cancer represents the major cause of cancer‐related deaths among men and ranks as the second leading cause of cancer among women worldwide.^1^ Based on histology, lung cancer consists of non–small‐cell lung cancer (NSCLC) and small‐cell lung cancer (SCLC).^2^ NSCLC accounts for 83% of total lung cancer and can be further divided into squamous carcinoma, adenocarcinoma and large cell carcinoma.^3^, ^4^ The main risk factors for NSCLC are cigarette smoke and air pollution.^5^, ^6^ Surgical resection is an effective approach in maintaining long‐term survival and thus widely used in lung cancer. In spite of the significant improvement in surgery and chemotherapy, the prognosis of metastatic lung cancer is still unsatisfactory, and the 5‐year survival rate for patients with NSCLC is lower than 21%.^7^ Therefore, favourable biomarkers are of great importance in the prediction of early metastasis of patients with lung cancer.

MicroRNAs (miRNAs), a group of small non‐coding RNAs, contribute to regulate gene expression at the posttranscriptional level through perfect or partial base‐pairing, mainly at the 3′UTR of the target messenger RNA. MiR‐326, for instance, was first identified among a set of miRNAs with remarkable high expression in neurons.^8^ It has been implicated that abnormal miR‐326 expression participates in a variety of pathological process, including multiple sclerosis, endometrial cancer, gastric cancer, lung cancer, osteosarcoma, pulmonary fibrosis and breast tumours.^9^, ^10^, ^11^, ^12^, ^13^, ^14^, ^15^, ^16^, ^17^, ^18^, ^19^, ^20^, ^21^ Of note, recent evidence revealed that miR‐326 regulated HbF synthesis by targeting erythroid Krüppel‐like factor (KLF), indicating the targeting effect of miR‐326 on KLF.^9^ Alternatively, miR‐326 reverses chemoresistance in human lung adenocarcinoma cells by targeting specificity protein 1 (Sp1).^12^

Krüppel‐like factor 3 (KLF3/BKLF), a member of the KLF family, was initially cloned from erythroid cells in a screen for factors with homology to the DNA‐binding domain of EKLF.^22^, ^23^ KLF3 encodes a sequence‐specific transcriptional repressor with three highly conserved C‐terminal C2H2 zinc finger motifs that bind CACCC boxes and other GC‐rich elements in the promoter and enhancer of target genes.^23^, ^24^ The N‐terminal domains are less conserved, and individual KLFs integrate different regulators to function as transcriptional activators or repressors.^25^ For example, recruitment of the acetyltransferases p300/CBP by KLF1 potentiates its activation of the *β‐globin* gene.^26^ However, KLF3 functions as a transcriptional repressor via recruitment of C‐terminal binding proteins 1 and 2 (CtBP1/2),^27^, ^28^ which in turn facilitates assembly of a potent silencing complex that drives epigenetic modification of gene regulatory regions.^25^, ^27^, ^28^, ^29^ Moreover, KLF3 is widely expressed and involved in multiple processes, such as erythropoiesis, metabolism and B lymphopoiesis.^25^, ^30^, ^31^, ^32^, ^33^, ^34^, ^35^ Although the roles of KLF3 have been broadly investigated in developmental processes, little is known about the function of KLF3 in lung cancer progression.

Specificity protein 1 (Sp1) is a transcription factor that is ubiquitously expressed in many mammalian cell types.^36^ Clinical evidences showed that Sp1 was overexpressed in a number of human cancers, such as gastric cancer,^37^ pancreatic adenocarcinoma^38^ and hepatocellular carcinoma.^39^ Emerging evidences showed that Sp1 accelerates tumour growth and metastasis through regulating gene transcription.^40^, ^41^ For example, overexpression of Sp1 increased the invasiveness of glioma cells via upregulating the expression and activity of MMP‐2.^42^ Choi et al also reported that Sp1 could bind to the promoter region of Slug and repressed EMT process.^43^ However, given many potential physiologic roles of Sp1, the target genes of Sp1 remain to be clearly characterized. Whether Sp1 could affect the expression of KLF3 in lung cancer has never been reported. Our preview studies have revealed that miR‐326 overexpression inhibited cell proliferation and migration in lung cancer.^44^ The potential targets, KLF and Sp1, were previously reported involved in the growth of lung cancer cells. 45, 46, 47 In this study, we aim to explore the possibility as well as related mechanisms of miR‐326‐Sp1‐KLF3 axis in the regulation of lung cancer development.

## 2 MATERIALS AND METHODS

For detailed Materials and methods, please see Supporting Information. For related sequences in the experiment please see Tables 1, 2, 3.

## 3 RESULTS

### 3.1 KLF3 is overexpressed in human lung cancer tissues

**Figure 1:**
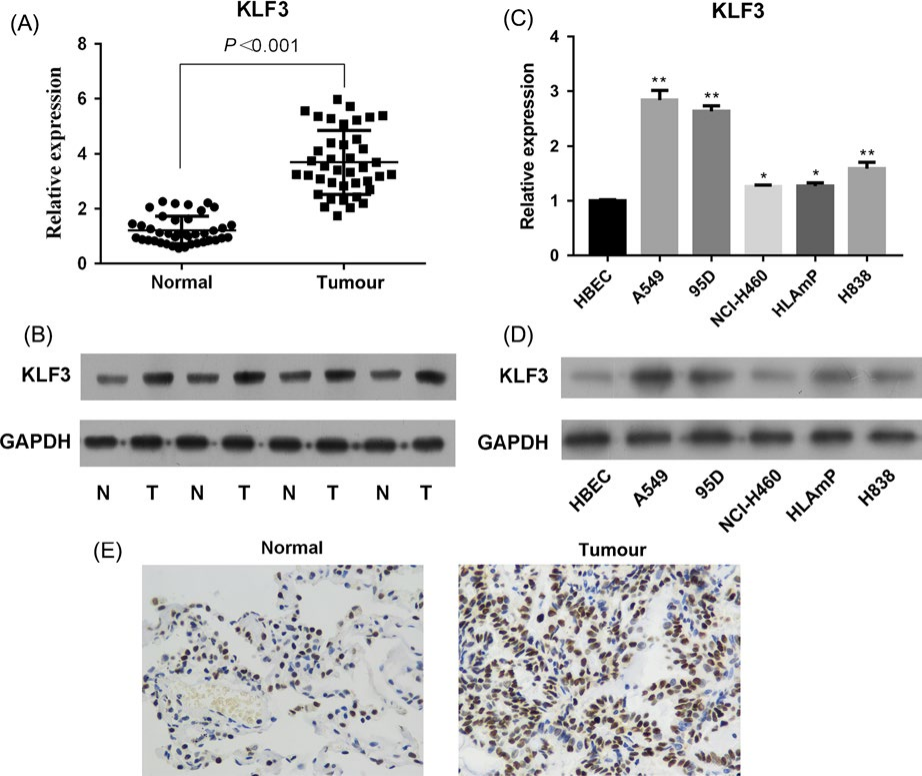
KLF3 is overexpressed in lung cancer tissues and cell lines. A, The mRNA level of KLF3 was measured by real‐time PCR. The results showed that KLF3 was upregulated in lung cancer tissues compared with paired adjacent normal tissues (N = 40), *P* < 0.001. B, Western blot analysis revealed that KLF3 was upregulated in lung cancer tissues. C and D, The mRNA and protein level of KLF3 was measured by real‐time PCR and Western blot in the human bronchial epithelial cell line HBEC and various lung cancer cell lines (A549, 95D, NCI‐H460, HLAmp and H838). E, Immunohistochemical analysis indicated that KLF3 was highly expressed in lung cancer tissues compared to that in normal tissues (×400). *indicates that *P* < 0.05 vs HBECs. **indicates that *P* < 0.001 vs HBECs

To explore the potential role of KLF3 in lung cancer progression, we firstly assessed the expression level of KLF3 in lung cancer tissues. Forty lung cancer tissues and paired tumour‐adjacent normal tissues were collected from Jiangsu Province Hospital, and the mRNA level of KLF3 was examined by real‐time PCR. The results showed that KLF3 was overexpressed in lung cancer tissues compared with that in adjacent normal tissues (Figure 1A, *P* < 0.001). Furthermore, we performed Western blot to evaluate the protein level of KLF3 in lung cancer tissues. Similarly, the KLF3 expression was highly upregulated in lung cancer tissues compared to that in the adjacent normal control (Figure 1B). The result of IHC indicated that the amount of KLF3 protein in lung cancer tissues was remarkably increased compared to that in normal lung tissues (Figure 1E). Then, we examined the expression of KLF3 in different lung cancer cell lines by real‐time PCR and Western blot. As shown in Figure 1C and D, the mRNA and protein levels of KLF3 were also significantly higher in lung cancer cell lines than that in control HBECs, especially in A549 and 95D cells (*P* < 0.01). Together, these results suggested that the highly expressed KLF3 may be associated with lung cancer progression.

### 3.2 Effects of suppression of KLF3 on lung cancer cells

**Figure 2:**
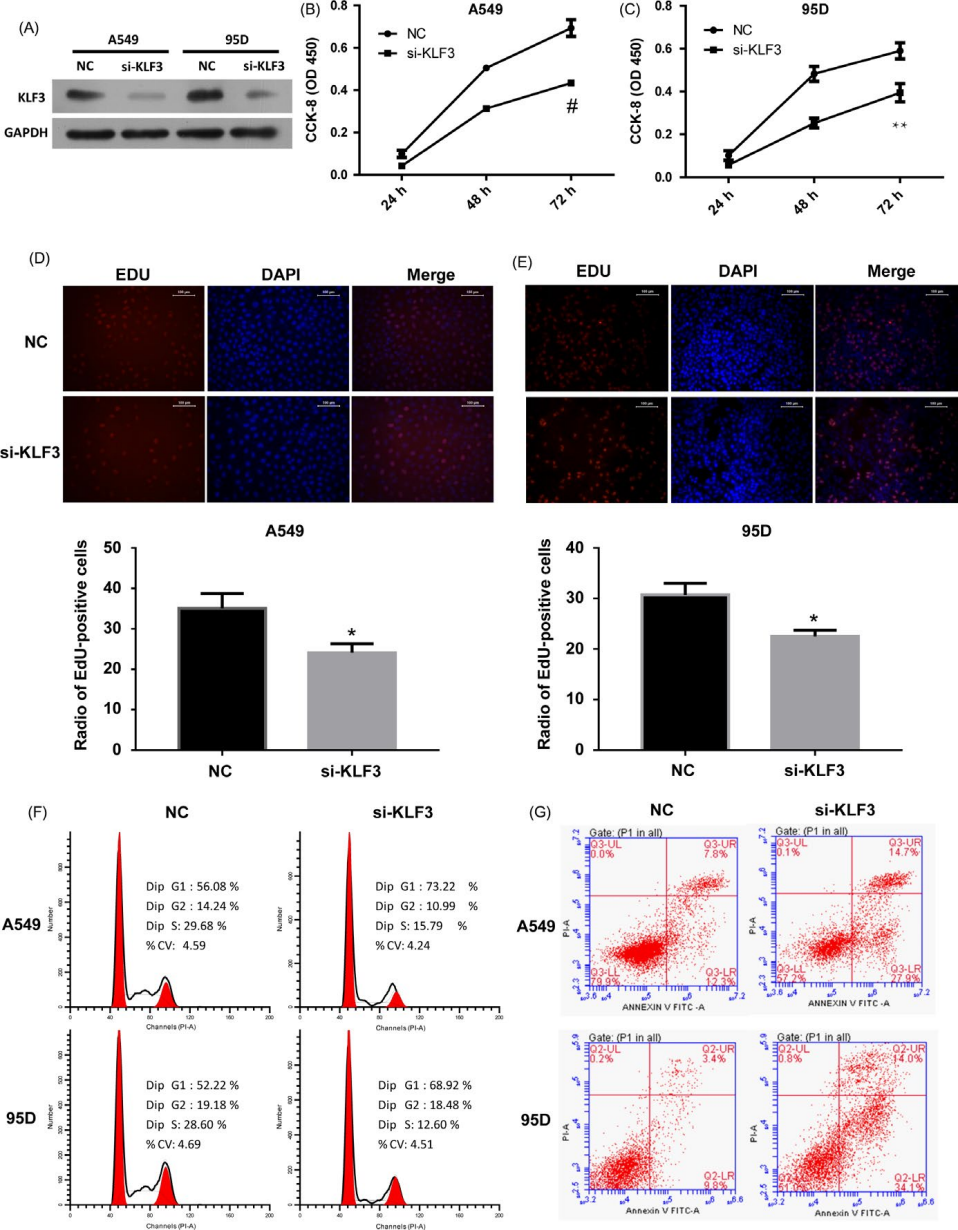
Effects of KLF3 downregulation on lung cancer cells. (A) The knockdown efficiency of KLF3 in lung cancer cells was confirmed by Western blot. (B and C) Cell proliferation was evaluated by CCK‐8 assay. The results showed that downregulate the expression of KLF3 inhibited cell proliferation in A549 and 95D cells. Experiments were performed three times; Mean ±SD. # indicates that *P* < 0.001; **indicates that *P* < 0.01. (D and E) EDU staining also revealed that silencing the expression of KLF3 inhibited cell proliferation in A549 (D) and 95D cells (E) (×200). Quantitative analysis of EDU‐positive A549 and 95D cells were shown as histograms. *indicates that *P* < 0.05. (F) Cell cycle analysis was performed by flow cytometry. Knockdown the expression of KLF3 resulted in cell cycle arrest at G1 phase. (G) Cell apoptosis was stained with PI and Annexin V double staining and analysed by flow cytometry. Statistical analysis revealed that siRNA‐mediated silencing of KLF3 induced cell apoptosis in A549 and 95D cells

To better understand the functional significance of KLF3 in regulating the biological processes of lung cancer cells, we performed loss‐of‐function experiments of KLF3 by transfection with specific siRNA targeting KLF3. The transfection efficiency was confirmed by Western blot in Figure S2, and the results showed that siRNA targeting KLF3 successfully downregulated the expression of KLF3 in both A549 and 95D cells (Figure 2A). Next, the proliferations of A549 and 95D cells were assessed by CCK‐8 assay. As shown in Figure 2B, downregulation of KLF3 significantly inhibited cell proliferation of A549 cells compared with the negative control (NC) cells (*P* < 0.001). Similar results were also obtained in 95D cells (Figure 2C, *P* < 0.01). Furthermore, we performed 5‐ethynyl‐2′‐deoxyuridine (EDU) staining to assess cell proliferation ability at 48 hours after transfection of KLF3 siRNA. Figure 2D and E also validated that knockdown of KLF3 inhibited cell proliferation in A549 and 95D cells (*P* < 0.05), consistent with the results obtained from CCK‐8 assay.

In order to investigate the mechanisms underlying the effect of KLF3 on cell proliferation of lung cancer cells, we examined cell cycle and apoptosis by flow cytometry. We observed that KLF3 knockdown increased the proportion of cells in the G1 phase and decreased the cells in S phase compared with the control (Figure 2F), suggesting that KLF3 could accelerate the cell cycle during lung cancer progression. Cell apoptosis was determined by using Annexin V‐fluorescein isothiocyanate (FITC) and propidium iodide (PI) double staining. The silencing of KLF3 induced cell apoptosis of A549 and 95D cells (Figure 2G), based on the observations of the percentage of the Annexin V‐positive cells (increased from 20.1% to 42.6% in A549 cells and 13.2% to 48.1% in 95D cells). Together, these results suggest that KLF3 facilitates the proliferation of lung cancer cells and may function as an oncogene during lung cancer progression.

### 3.3 Downregulation of KLF3 inhibited cell migration and invasion

**Figure 3:**
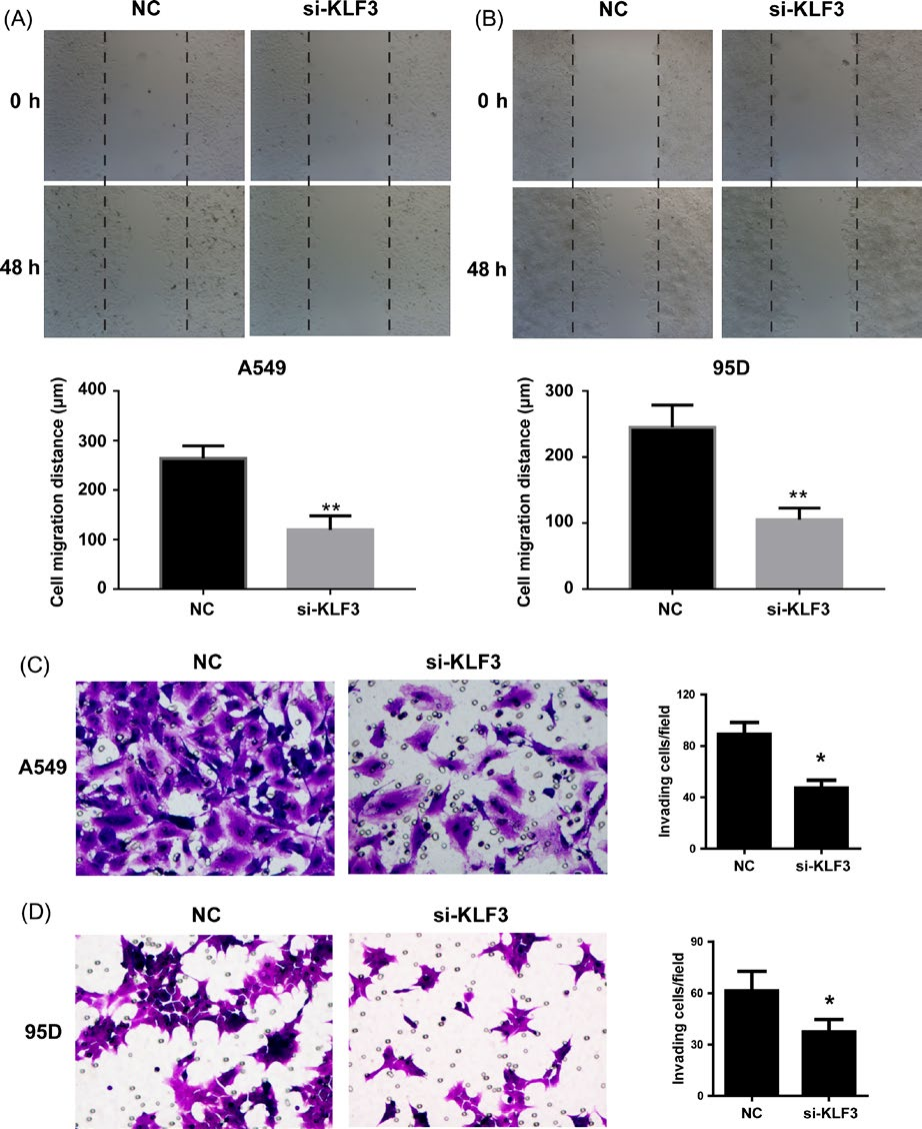
The decrease in KLF3 expression reduces cell migration and invasion. (A and B) Wound‐healing assay was used to detect cell migration ability in A549 and 95D cells. Compared with NC, si‐KLF3 cells showed weaker migration ability. Quantitative analysis of cell migration distance was shown as histograms. **indicates that *P* < 0.01. (C and D) Transwell assays showed that knockdown the expression of KLF3 significantly inhibited cell invasion in A549 (C) and 95D cells (D) (×200). Experiments were performed 3 times. Representative images of invaded cells were shown. Statistical analysis showed the effect of KLF3 on cell invasion. Mean ± SD. *indicates that *P* < 0.05

A critical feature of lung cancer is aggressive behaviour in distant metastasis sites including lymph node. Cell migration and invasion are important steps in the process of metastasis. To determine whether KLF3 affects inhuman lung cancer cell migration and invasion, wound healing and cell invasion assays were performed. The results showed that in untreated A549 cells, wound closure occurred gradually at 48 hours after scratch, whereas this effect was significantly reduced by the treatment of si‐KLF3 (Figure 3A). Similar results were obtained in 95D cells (Figure 3B). In line with this finding, suppression of KLF3 significantly repressed the invasive ability of A549 and 95D cells (*P* < 0.01) (Figure 3C and D). In summary, these results indicated that KLF3 participated in the regulation of lung cancer cell proliferation, migration and invasion.

### 3.4 The block of KLF3 suppressed the activation of JAK2/STAT3 and PI3K/AKT signalling pathways in vitro

**Figure 4:**
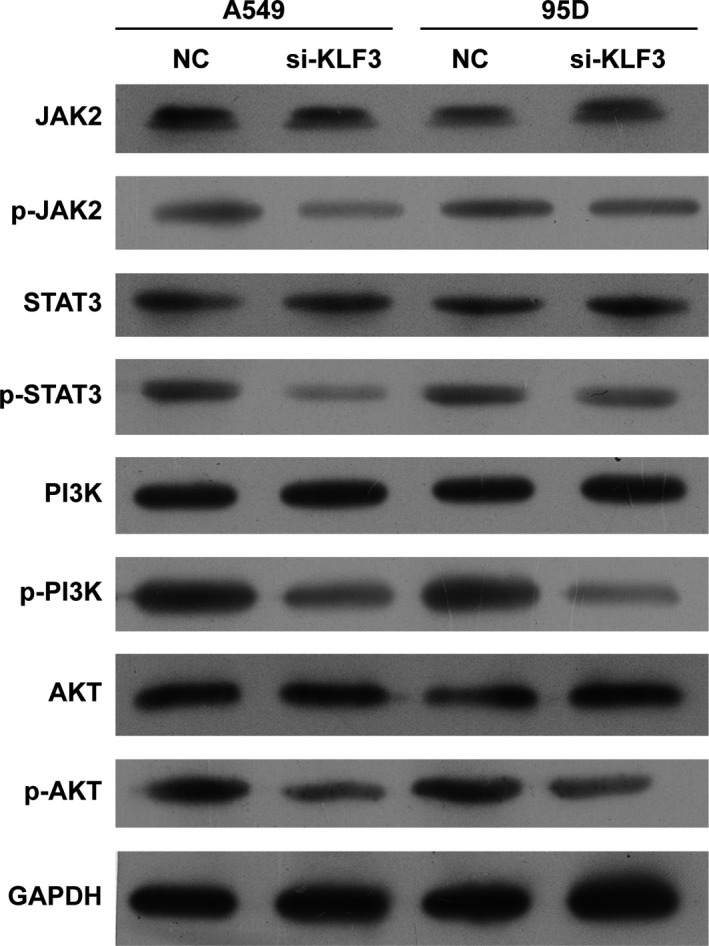
Knockdown of KLF3 suppressed the activation of JAK2/STAT3 and PI3K/AKT signalling pathways in vitro. Western blot analysis demonstrated that knockdown the expression of KLF3 significantly decreased the expression of JAK2, STAT3, PI3K, and AKT phosphorylation levels in A549 and 95D cells in vitro

Considering that the promoting effect of KLF3 on cell growth and migration, we performed Western blot assay to measure which signalling pathways were involved by KLF3. As revealed in Figure 4, the phosphorylation levels of JAK2, STAT3, PI3K and AKT were downregulated in the absence of KLF3 in both A549 and 95D cells in vitro, suggesting that JAK2/STAT3 and PI3K/AKT signalling pathways may be involved in lung cancer progression regulated by KLF3.

### 3.5 Sp1 is positively correlated with the expression of KLF3

**Figure 5:**
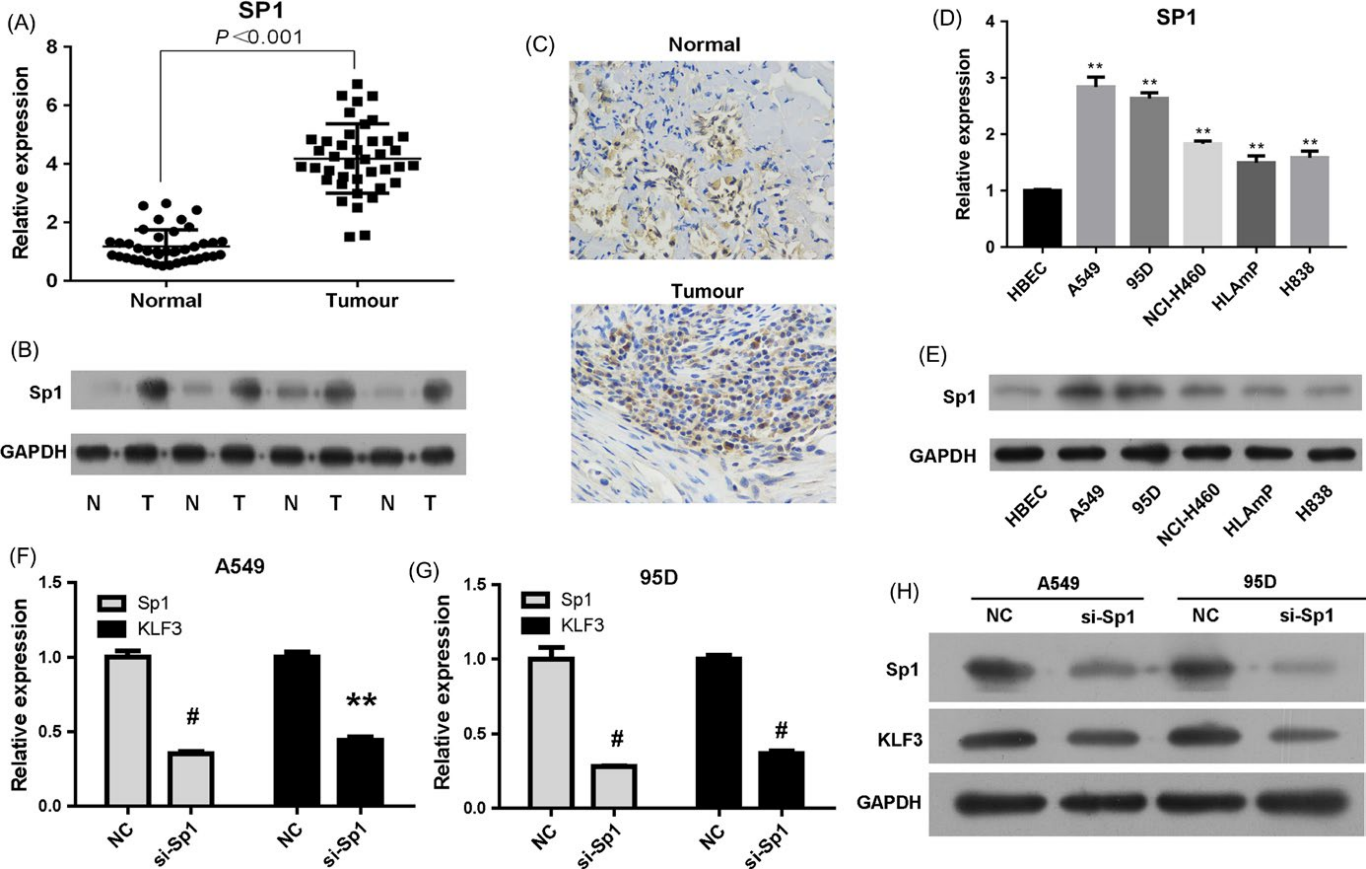
KLF3 expression is positively regulated by Sp1. A, The mRNA level of Sp1 was detected by real‐time PCR. Compared with the normal tissues, Sp1 was upregulated in lung cancer tissues, *P* < 0.001. B, Western blot analysis revealed that Sp1 was upregulated in lung cancer tissues. C, Immunohistochemical analysis indicated that the level of Sp1 was increased in lung cancer tissues compared to that in normal tissues (×400). D and E, The mRNA and protein levels of Sp1 were examined by real‐time PCR and Western blot. The results showed that Sp1 was overexpressed in lung cancer cell lines. ** indicates that *P* < 0.001 vs HBECs. F and G, Real‐time PCR results showed that KLF3 expression was downregulated after silencing the expression of Sp1 in A549 and 95D cells. Mean ± SD. **indicates that *P* < 0.01; # indicates that *P* < 0.001. H, Western blot analysis confirmed that knockdown the expression of Sp1 significantly reduced KLF3 expression

Based on the above findings, the function of KLF3 in promoting cell proliferation, migration and invasion have been well demonstrated. However, little is known about the regulatory mechanism of KLF3 in lung cancer cells. Through bioinformatics analysis of the proximal promoter of KLF3, we found that there are some putative binding sites of Sp1 (Figure S1). Therefore, we performed experiments to investigate the possible relation between KLF3 and Sp1. Firstly, we detected the expression of Sp1 in lung cancer tissues through real‐time PCR and Western blot. The results showed that Sp1 was upregulated in lung cancer tissues compared with that in the adjacent normal tissues (Figure 5A and B, *P* < 0.001), which correlated with the expression of KLF3 in lung cancer tissues (Figure 1A and B). The IHC data also indicated that the level of Sp1 in lung cancer tissues was evidently elevated compared to that in normal lung tissues (Figure 5C). As expected, the mRNA and protein levels of Sp1 were also elevated in different lung cancer cell lines compared with that in the HBECs (Figure 5D and E). Next, we examined the expression of KLF3 after reducing of Sp1 level in A549 and 95D cells. Figure 5F showed that the mRNA level of KLF3 was downregulated after downregulation of Sp1 by siRNA in A549 cells compared with that in the NC group (*P* < 0.01). Similarly, the mRNA expression of KLF3 was also decreased in 95D cells (Figure 5G, *P* < 0.01). Moreover, Western blot analysis revealed that the protein level of KLF3 was obviously decreased after expression of Sp1 declined in A549 and 95D cells (Figure 5H). These data indicated Sp1 positively correlated with the expression of KLF3 in lung cancer cells.

### 3.6 KLF3 is a direct target of Sp1

**Figure 6:**
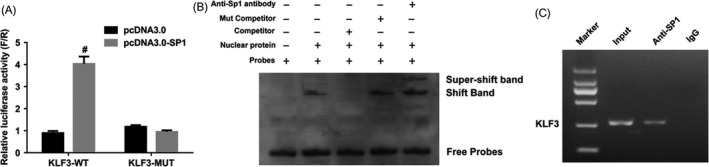
KLF3 is a direct target gene of Sp1. A, The luciferase activity of KLF3 was detected by Dual‐luciferase report assay after transfection of the WT and Mutant constructs alone or together with the Sp1 expression vector. The results showed that KLF3 promoter activity was significantly enhanced by Sp1. #indicates that *P* < 0.001. B, EMSAs were used to investigate the binding of Sp1 and KLF3 promoter in vitro. Nuclear protein was extracted from A549 cells. When DNA probes were incubated with nuclear extracts, a specific DNA‐protein complex was observed and was supershifted by the anti‐Sp1 antibody. C, ChIP analysis of Sp1 at the KLF3 promoter region in A549 cells. The results suggested that Sp1 can directly bind to the promoter region of KLF3

To further explore the molecular basis for this regulation, we amplified the promoter region of KLF3 and performed dual‐luciferase reporter assay. The wild‐type (WT) and mutant promoters were transfected alone or with the Sp1 expression vector into HEK293T cells. The results showed that overexpression of Sp1 markedly increased the relative luciferase activity of KLF3‐WT (Figure 6A, lanes 1 and 2), but it did not activate the relative promoter activity of the mutant KLF3 (Figure 6A, lanes 3 and 4), suggesting the interaction between promoter region of KLF3 and Sp1 putative binding sites. To further determine whether KLF3 is a direct target gene of Sp1, we performed electrophoretic mobility shift assays (EMSAs) in vitro using the nuclear extracts from A549 cells and the oligonucleotide probes containing the predicted Sp1 binding sites. The results demonstrated that shift bands were observed when probes were incubated with nuclear extracts (Figure 6B). Moreover, as expected, super‐shift bands were observed when Sp1 antibody was added to the binding system (Figure 6B). In addition, chromatin immunoprecipitation (ChIP) assay was initially employed to further determine whether Sp1 could bind to the promoter of KLF3. The pull‐down DNA was identified by RT‐PCR and the results showed that KLF3 was markedly amplified from the Sp1‐immunoprecipitated A549 chromatins, but absent from chromatins immunoprecipitated by IgG (Figure 6C). In summary, these results demonstrate that KLF3 is a direct target gene of Sp1.

### 3.7 Sp1 is a downstream target of miR‐326

**Figure 7:**
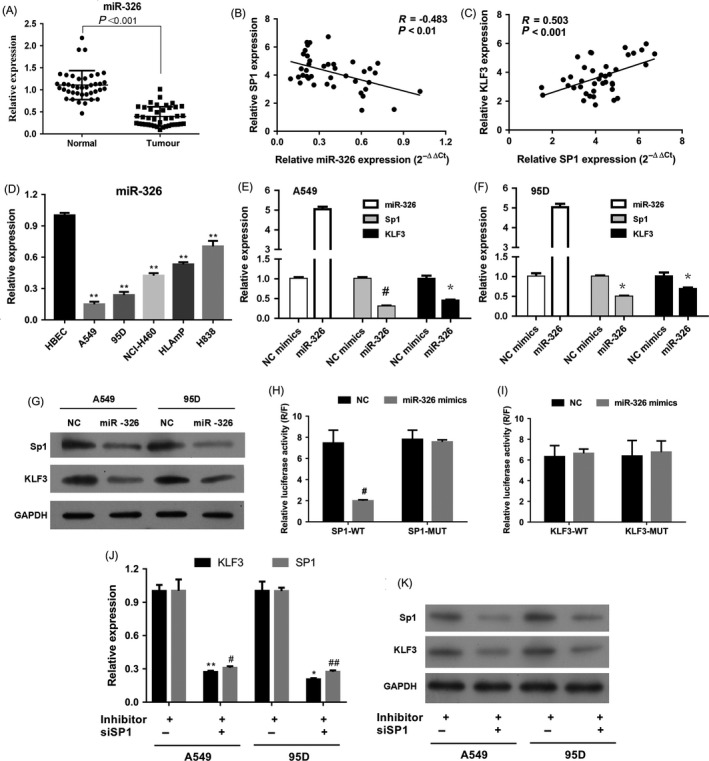
Sp1 is a direct downstream target of miR‐326. A, Relative expression of miR‐326 in lung cancer tissues was confirmed by real‐time PCR. N = 40. *P* < 0.001. B, Correlation analysis between miR‐326 and Sp1 levels. *P* < 0.01. C, Correlation analysis between Sp1 and KLF3 expressions. *P* < 0.001. D, Real‐time PCR results revealed that miR‐326 was downregulated in lung cancer cell lines. **P < 0.001 vs HBECs. E, Overexpression of miR‐326 downregulated the expression of Sp1 and KLF3 in A549 cells. Mean ± SD. *P < 0.05; #*P* < 0.05. F, Overexpression of miR‐326 reduced the expression of Sp1 and KLF3 in 95D cells. Mean ± SD. *P < 0.05; #*P* < 0.05. G, Western blot analysis demonstrated that miR‐326 inhibited Sp1 and KLF3 expression in A549 and 95D cells. H, Luciferase activity of Sp1 wild type (wt) was inhibited by using miR‐326 mimics, compared to Sp1 mutant (mut). #*P* < 0.05. I, No significant difference of luciferase activity of KLF3 wild type (wt) and KLF3 mutant (mut) was found by using miR‐326 mimics. #*P* > 0.05. J, The levels of Sp1 and KLF3 mRNAs were significantly reduced in group of miR‐326 inhibitor+siSP1 than those of miR‐326 inhibitor+siRNA‐NC in both A549 and 95D cells. miR‐326 antagomir was used as inhibitor. *P < 0.05; #*P* < 0.05; **P < 0.01; ##*P* < 0.01. K, The expressions of Sp1 and KLF3 proteins were obviously decreased in group of miR‐326 inhibitor+siSP1 than those of miR‐326 inhibitor+siRNA‐NC in both A549 and 95D cells. miR‐326 antagomir was used as inhibitor

In our preview studies, we have found that miR‐326 inhibited lung cancer cell proliferation and migration.^44^ Meanwhile, through bioinformatics analysis such as JASPAR and TargetScan, we found the 3′UTR regions of KLF3 and Sp1 contained the putative binding sites of miR‐326, suggesting Sp1 and KLF3 may be potential downstream targets of miR‐326. To verify the prediction, we firstly examined the level of miR‐326 in lung cancer tissues and cell lines. As shown in Figure 7A, compared with the normal tissues, miR‐326 was significantly downregulated in lung cancer tissues. We further analysed the relations between Sp1, KLF3 and miR‐326 and found that the level of miR‐326 was significantly negatively correlated with Sp1 expression (*R* = −0.483, *P* < 0.01) but positively with KLF3 level (*R* = 0.503, *P* < 0.001) (Figure 7B, C). Based on this finding, we then detected the miR‐326 level in distinct types of cells and the data indicated that the expressions in lung cancer cell lines were statistically reduced that in HBECs (Figure 7D). Furthermore, to investigate whether miR‐326 could regulate Sp1 and KLF3 expression, we transfected miR‐326 mimics into lung cancer cells and real‐time PCR results revealed that overexpression of miR‐326 suppressed the expression of Sp1 and KLF3 in both A549 (Figure 7E) and 95D (Figure 7F) cells. Similarly, Western blot analysis also showed that the expression of Sp1 and KLF3 was markedly deterred due to miR‐326 upregulation (Figure 7G). We then employed dual‐luciferase reporter assays to verify the direct relationship. The results showed that transfection of miR‐326 mimics significantly decreased the luciferase activity of Sp1 3′UTR compared with cells transfected with NC, whereas the effect was abolished when the binding sites of miR‐326 were mutant (Figure 7H). However, the no significant change regarding luciferase activity of KLF3 3′UTR was found by using miR‐326 mimics, suggesting KLF3 was not a direct gene of miR‐326 (Figure 7I). In order to further validate the miR‐326/Sp1/KLF3 regulatory axis, we impeded the expression of Sp1. Notably, the KLF3 level was remarkably inhibited on account for the overexpression of miR‐326 (Figure 7E‐G). The downregulation of miR‐326 thus induced the level of KLF3, but this can be reversed by suppression of Sp1, which suggests that miR‐326 downregulation increases the KLF3 overexpression through the upregulation of Sp1 (Figure 7J, K).

### 3.8 miR‐326 and KLF3 are associated with overall survival in patients with lung cancer

**Figure 8:**
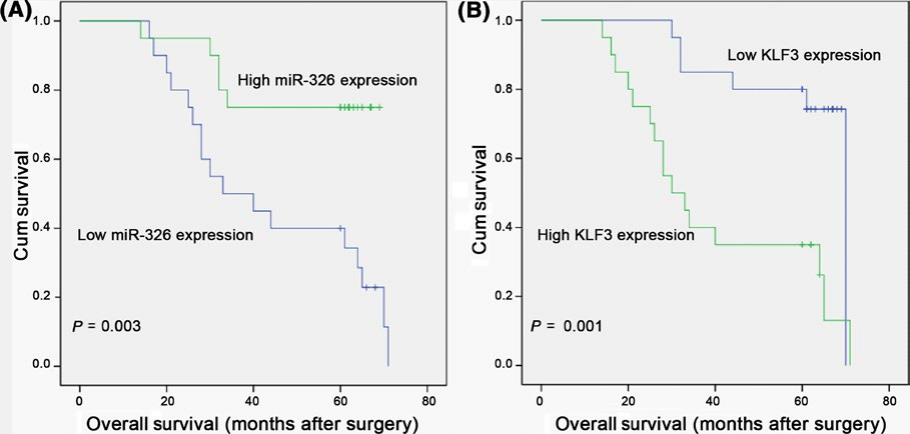
miR‐326 and KLF3 are associated with overall survival (OS) in patients with lung cancer. A, Kaplan–Meier plots of OS in patients with lung cancer, stratified by miR‐326 expression. The *P* value was calculated by a log‐rank test. B, Kaplan–Meier plots of OS stratified by KLF3 expression. The results showed that high levels of KLF3 were associated with significantly poorer OS in patients with lung cancer, *P* = 0.001

Based on the above findings, we further evaluated whether miR‐326 and KLF3 expression are associated with clinical prognosis. Through analysis of a dataset of lung cancer that includes 40 patients, we found that decreased miR‐326 expression was associated with poor prognosis among patients with lung cancer (Figure 8A, *P* = 0.003). Of note, the patients with high level of KLF3 were associated with poor overall survival (OS) caused by lung cancer (Figure 8B, *P* = 0.001). Clinicopathologic features were compared between these groups (Table 4), and significantly higher recurrence as well as death rate was observed in lung cancer patients with low miR‐326 and high KLF3 levels, when compared to the high‐miR‐326 group and the low‐KLF3 group (*P* < 0.05). However, the miR‐326 and KLF3 expression levels were not associated with age (*P* = 0.093 and *P* = 0.069, respectively), gender (*P* = 0.05) and TNM tumour stage (*P* = 0.278 and *P* = 0.207, respectively). These findings indicate that miR‐326/Sp1/KLF3 regulatory axis participated in lung cancer progression.

**Table 1:** Sequences of relative primers

ID	Forward sequence (5′‐3′)	Reverse sequence (5′‐3′)
U6	CTCGCTTCGGCAGCACA	AACGCTTCACGAATTTGCGT
GAPDH	CCTCGTCTCATAGACAAGATGGT	GGGTAGAGTCATACTGGAACATG
miR‐326	ACACTCCAGCTGGGCCTCTGGGCCCTTCCTCC	CTCAACTGGTGTCGTGGAGTCGGCAATTCAGTTGAGCTGGAGG
KLF3	GCCTCTCATGGTCTCCTTATC	TAATCTGTCCTCTGTGGTTCG
SP1	TGGCAGCAGTACCAATGGC	CCAGGTAGTCCTGTCAGAACTT

### 3.9 The decrease of KLF3 inhibited the growth of xenograft tumour in nude mice

**Figure 9:**
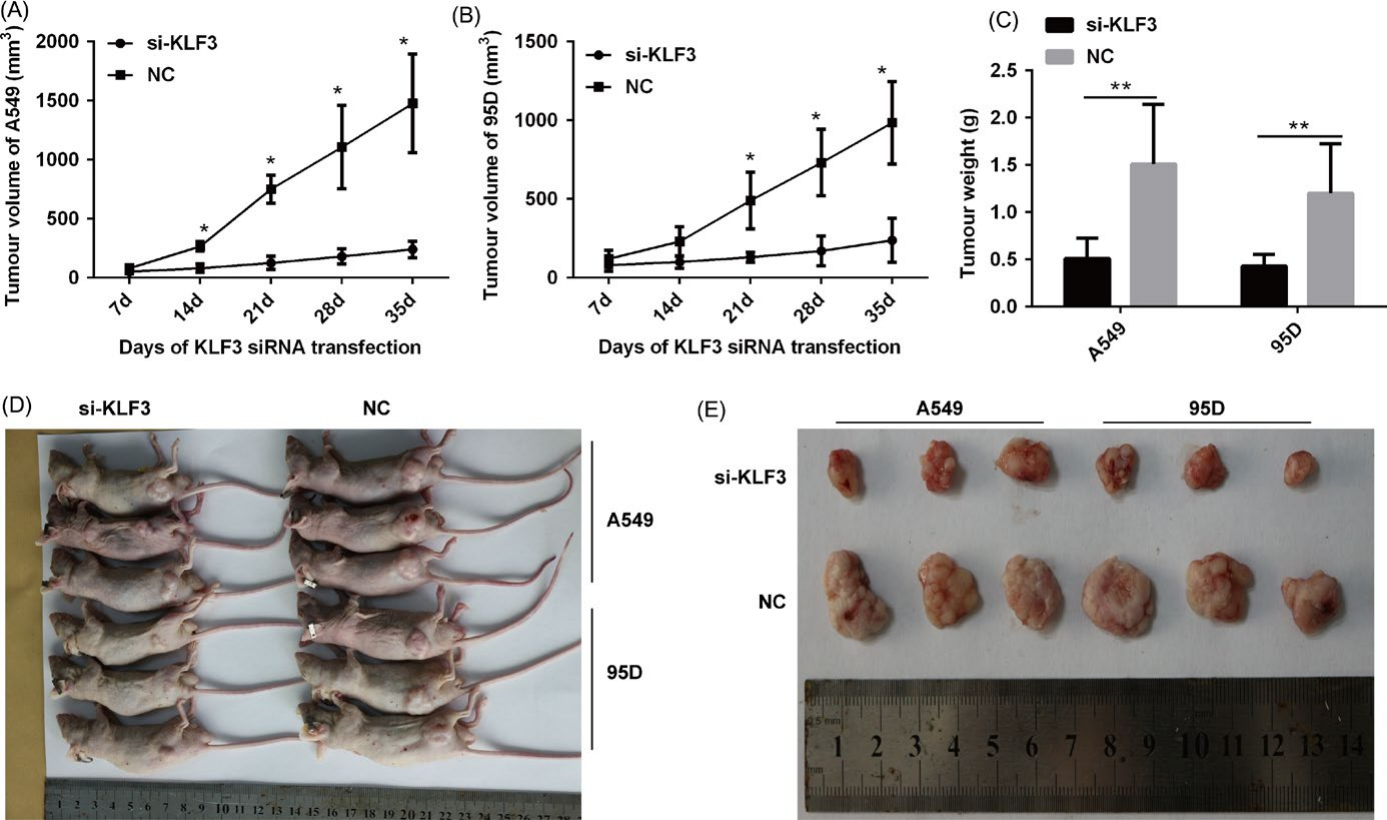
Knockdown of KLF3 inhibited the growth of xenograft tumour in nude mice. (A and B) Line graphs showed that knockdown the expression of KLF3 significantly inhibited the tumour volume in nude mice injecting A549 (A) and 95D cells (B). *indicates that *P* < 0.05. (C) Bar graph demonstrated that knockdown the expression of KLF3 remarkably suppressed the tumour weight in nude mice injecting A549 and 95D cells. **indicates that *P* < 0.01. (D and E) Gross observation of xenograft tumour revealed that the tumour volume in nude mice injecting KLF3‐silenced A549 and 95D cells was much smaller

**Figure 10:**
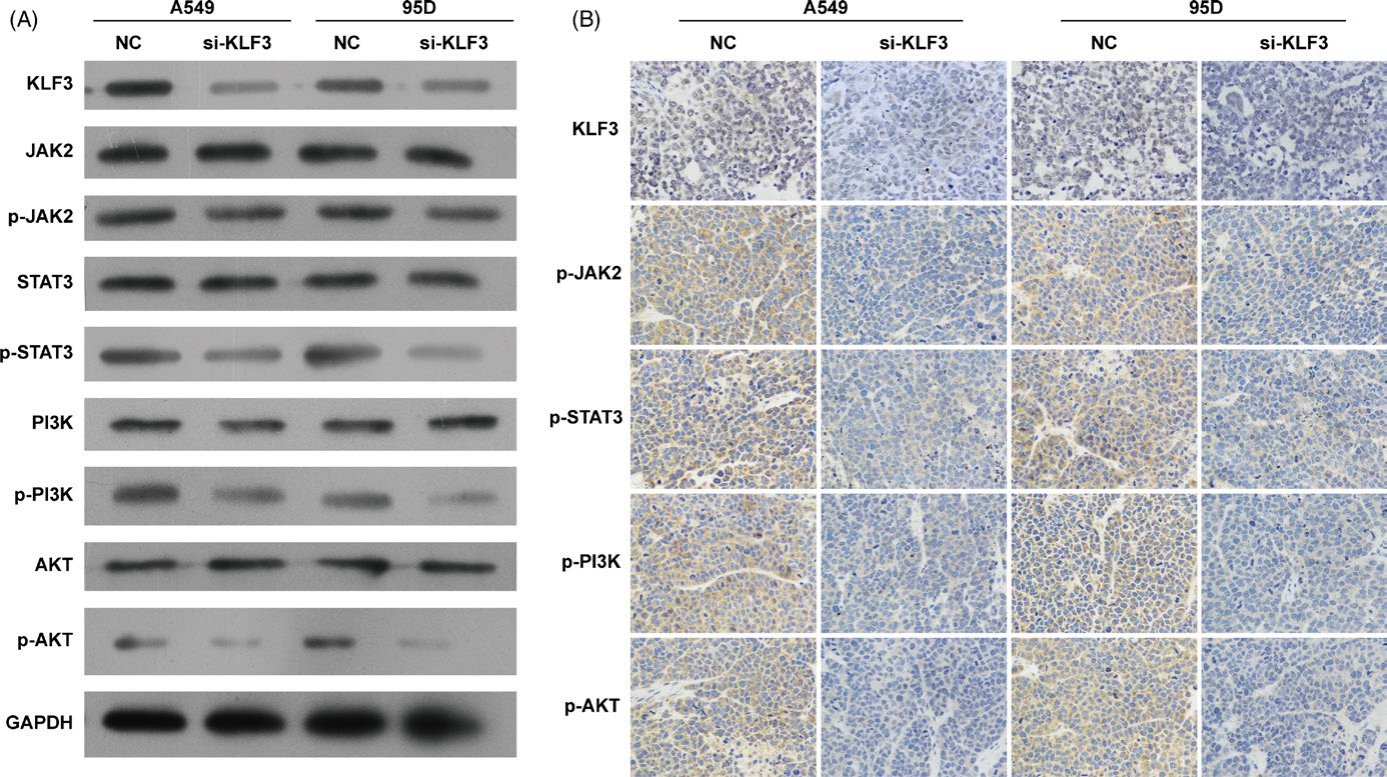
Knockdown of KLF3 suppressed the activation of JAK2/STAT3 and PI3K/AKT signalling pathways in vivo. A and B, Western blot and immunohistochemical analysis (×400) revealed that knockdown the expression of KLF3 obviously downregulated the phosphorylation levels of JAK2, STAT3, PI3K, and AKT proteins in nude mice injecting KLF3‐silenced A549 and 95D cells in vivo

To further investigate the effect of KLF3 on tumour growth in vivo, both A549 and 95D cells were transfected with NC or si‐KLF3 and then hypodermically injected into the BALB/c nude mice. The reduction in KLF3 significantly depressed the tumour volumes in a time‐dependent manner (*P* < 0.05) and also remarkably lowered the tumour weights in both A549 and 95D cells (Figure 9A‐E, *P* < 0.05). Consistent with the results in vitro, Western blot and immunohistochemical assays revealed that the phosphorylation levels of JAK2, STAT3, PI3K and AKT proteins also declined in BALB/c nude mice injecting A549 and 95D cells with decreasing level of KLF3 (Figure 10A and B). Together, these results suggest that KLF3 is involved in the tumour growth of A549 and 95D cells through regulating JAK2/STAT3 and PI3K/AKT signalling pathways in vivo.

**Table 2:** siRNA sequences

Name	Sense (5′‐3′)	Antisense (3′‐5′)
Negative control	UUCUCCGAACGUGUCACGUTT	ACGUGACACGUUCGGAGAATT
siKLF3‐534	GCAUGCAAGUACCUGUAAUUU	UUCGUACGUUCAUGGACAUUA
siKLF3‐772	GAUACACAGAUGUGAUUAUUU	UUCUAUGUGUCUACACUAAUA
siKLF3‐902	GUCUGAUGAACUAACAAGAUU	UUCAGACUACUUGAUUGUUCU
siSp1‐491	CCUGGAGUGAUGCCUAAUAUU	UUGGACCUCACUACGGAUUAU
siSp1‐996	CCAGCAACAUGGGAAUUAUUU	UUGGUCGUUGUACCCUUAAUA
siSp1‐1648	GCCGUUGGCUAUAGCAAAUUU	UUCGGCAACCGAUAUCGUUUA

**Table 3:** Sequences of relative primers for recombinant vector construction

ID	Sequence (5′‐3′)
SP1‐XhoI F	CCGCTCGAGGATCAGGCACCCGGGG
SP1‐NotI R	ATTTGCGGCCGCCAGCCAACCCCTGTGAATG
SP1 MUT‐F	GATCAGGCACCCGGGGTTCACATCATATGGGCCATACC
SP1 MUT‐R	GGTATGGCCCATATGATGTGAACCCCGGGTGCCTGATC
KLF3‐KpnI ‐F	GGGGTACCTTCCCGCTGGAGCCG
KLF3‐XhoI R	CCGCTCGAGTGCCCGCTCCGGG
KLF3 MUT‐F	CCTGGCAGCCCGCGCTATAACGGCTTGCTCGCCCGCGAGCC
KLF3 MUT‐R	GGCTCGCGGGCGAGCAAGCCGTTATAGCGCGGGCTGCCAGG

**Table 4:** Correlation between miR‐326 and KLF3 mRNA expression and clinicopathological characteristics with lung cancer

Parameter Clinical	Overall	Expression of miR‐326			Expression of KLF3		
		Low	High	*P* value	Low	High	*P* value
In Total	40	20 (50.00)	20 (50.00)	‐	20 (50.00)	20 (50.00)	‐
Age (y, mean ± SD)	63.90 ± 9.98	61.25 ± 9.59	66.55 ± 9.88	0.093	67.55 ± 9.71	60.25 ± 9.05	0.069
Male (n, %)	33 (82.50)	17	16	0.5	16	17	0.5
Follow‐up duration (months, mean ± SD)	48.80 ± 19.33	42.65 ± 20.47	54.95 ± 16.37	0.043	58.60 ± 12.98	39.00 ± 19.90	0.001
TNM (n, %)							
IB	17 (42.50)	6	11	0.278	11	6	0.207
IIA	10 (25.00)	6	4		3	7	
IIB	13 (32.50)	8	5		6	7	
Recurrence (n, %)							
Overall	23 (57.50)	18	5	<0.001	7	16	0.01
1‐y recurrence	11 (27.50)	9	2	0.031	1	10	0.003
5‐y recurrence	12 (30.00)	9	3	0.082	6	6	1
Death	22 (55.00)	17	5	<0.001	6	16	0.004
5‐y actual survival	23 (57.50)	5	12	0.054	13	4	0.01

## 4 DISCUSSION

KLF3 was originally discovered as a CACCC box‐binding transcription factor in erythroid cells.^48^ In fact, similar to other KLF members, KLF3 exhibits both transcriptional activation and repression via interaction with other factors.^27^, ^49^ Although the role of KLF3 has been extensively studied in the development of hematopoietic system, little is known about KLF3 in cancer. In 2011, Magali et al reported that the expression of KLF3 was downregulated in patients with acute myeloid leukaemia compared with healthy donors.^50^ In addition, Sachdeva et al found that KLF3 expression was downregulated in both primary mouse and human metastatic sarcomas due to the promoter hypermethylation.^51^ Intriguingly, increased Krüppel‐like factor 6 splice variant 1 (KLF6‐SV1) expression is associated with poor prognosis in prostate, lung and ovarian cancer.^52^ By contrast, the mRNA expression level of Krüppel‐like factor 6 splice variant 2 (KLF6‐SV2) in colorectal cancer tissues was decreased than in corresponding normal tissues.^53^ KLF6 expression is also decreased in gastric cancers. It has been implicated that the difference of KLF3 expression is owing to the loss of heterozygosity, mutations and alternative splicing. 54, 55 Identically, the difference in expressional changes exists among various types of cancers. For instance, in our study, KLF3 expression was elevated in lung cancer tissues, and abnormal high level of KLF3 was associated with poor overall survival in patients with lung cancer, which was consistent with previous finding.^56^ The decrease in KLF3 reduced cell proliferation, migration and invasion in vitro, and also inhibited tumour growth in BALB/c nude mice, suggesting that KLF3 may function as an oncogene during lung cancer progression. Nevertheless, KLF3 was downregulated in acute myeloid leukaemia and sarcomas,^50^, ^51^ and loss of KLF3 was correlated with aggressive phenotypes and poor survival outcomes in colorectal cancer.^57^ Additionally, downregulation of KLF3 blocked JAK2/STAT3 and PI3K/AKT signalling pathways. It is generally known that aberrant JAK2/STAT3 and PI3K/AKT signalling pathways are associated with the occurrence and development of cancers including tumour cell proliferation, migration and apoptosis.^58^, ^59^, ^60^ Thus, we propose KLF3 may mediate JAK2/STAT3 and PI3K/AKT signalling pathways during lung cancer development. The distinct splicing, differential enrichment of KLF3 and leading pathways in diverse organs may result in contrasting level of KLF3 in the development of tumours.

As a transcription factor, KLF3 was already known to exert as a strong transcriptional repressor. Some studies have identified the potential regulatory mechanisms of KLF3 in different tissues. Dewi et al found that phosphorylation of KLF3 and CtBP2 by HIPK2 strengthens the interaction between these two factors and increases transcriptional repression by KLF3.^29^ KLF3 can also be modified by small ubiquitin‐like modification (SUMOylation).^61^ Lack of SUMOylation and disconnection with CtBP result in a loss of transcription repression and gain of transcription activation potential.^61^ Through bioinformatics analysis, certain putative binding sites of Sp1 were found related to the promoter of KLF3, which suggests that Sp1 presents as a positive regulator of KLF3. Next, we examined the expression of KLF3 after impeding Sp1 level and found that KLF3 was downregulated in A549 and 95D cells. Finally, the luciferase reporter assay, EMSA and ChIP assays validated that KLF3 was a novel direct target gene of Sp1.

Sp1 is an ubiquitously expressed transcription factor and is considered to be a general transcription factor required for transcription of a large number of housekeeping genes.^40^, ^62^ However, accumulating evidence indicates that Sp1 is upregulated in various types of human cancer, including lung cancer.^63^ Consistent with these reports, our study also revealed that Sp1 was increased in lung cancer tissues compared with that in adjacent normal tissues.

Usually, miRNAs modulate cancer cell proliferation and migration by restricting expression of downstream targets. The interaction between lncRNA PDIA3P and miR‐185‐5p affects oral squamous cell carcinoma progression by targeting cyclin D2.^64^ Sp1 contributes to tumour progression and is regulated by many miRNAs. Mao et al demonstrated that miR‐330 inhibited prostate cancer cell migration by targeting Sp1.^65^ Wang et al also revealed that miR‐375 could bind to the 3′UTR of Sp1. In lung cancer, Sp1 could be repressed by miR‐29c, miR‐27b, miR‐335 and miR‐429 in translational level.^66^, ^67^, ^68^ Similar to these findings, our previous study revealed that miR‐326 was deterred in lung cancer cells, and transfection of miR‐326 significantly decreased cell proliferation and migration.^44^ Recently, the increasing studies have focused on the target genes of miR‐326 that could interact with miR‐326 to impede tumour development and progression, such as NOB1,^11^ phox2a,^44^ CCND1,^69^ FSCN1^15^ and NSBP1.^70^ In the present study, we demonstrated that Sp1 is a downstream target of miR‐326. We note that non‐coding RNAs, including lncRNA, miRNA, circRNA, as well as their interactions, play a pivotal role in regulation of various types of cancer and other diseases such as acute myocardial infarction, spinal muscular atrophy and cardiac dysfunction. 71, 72, 73, 74, 75, 76, 77, 78, 79, 80, 81 Particularly, long non‐coding RNA XIST acts as an oncogene in non–small‐cell lung cancer by epigenetically repressing KLF2 expression, indicating that lncRNAs also exert mediating function by targeting KLF protein.^75^ Our further study may focus the regulatory axis of lncRNA‐miRNA in the progression of lung cancer, as well as to elucidate the possible mechanisms.

In summary, our study provides evidence that miR‐326/Sp1/KLF3 regulatory axis involves in lung cancer cell proliferation, migration and invasion, which provide leads for the further therapy to patients with lung cancer.

## CONFLICT OF INTERESTS

None.

## AUTHORS’ CONTRIBUTION

RW, YQS and WD conceived the study and performed the whole experimental work in its design and coordination. WZ, JLX, TZQ, JL, MLZ, QQW and TPX participated in parts of the experiments and analysis of the data. JX, LJZ, RHG, KHL, YMY, YHG, PWH, PL and LKL contributed to provide clinical samples and parameters. The manuscript was written by RW. All authors read and approved the final manuscript.
