# Supplementary figures and images for: MiR‐326/Sp1/KLF3: A novel regulatory axis in lung cancer progression
Source: Cell Prolif. 2018 Nov 28;52(2):e12551. doi: 10.1111/cpr.12551 (PMC6495967; doi:10.1111/cpr.12551)

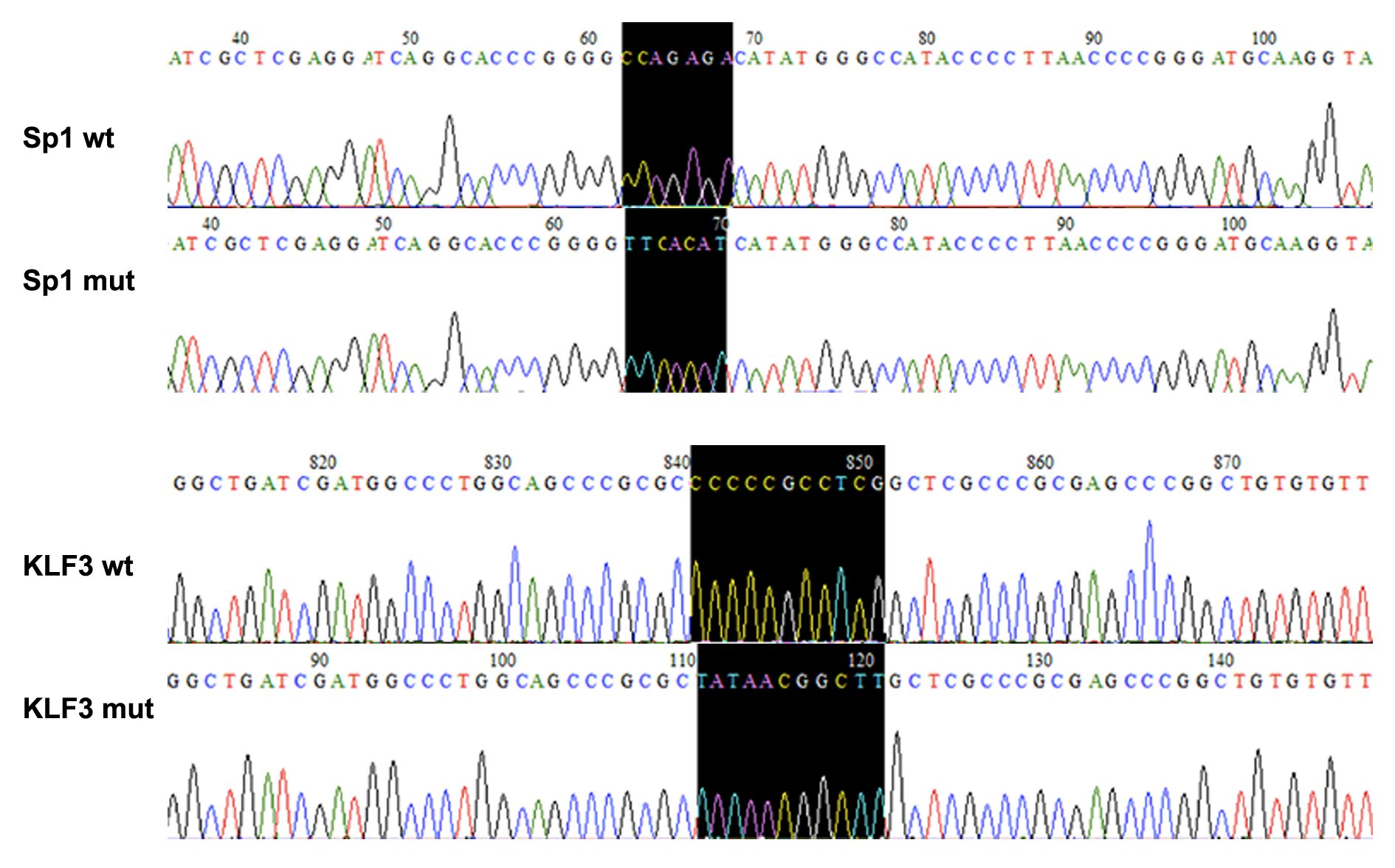

Supplement: Supplementary file 1 [file CPR-52-e12551-s001.tif]

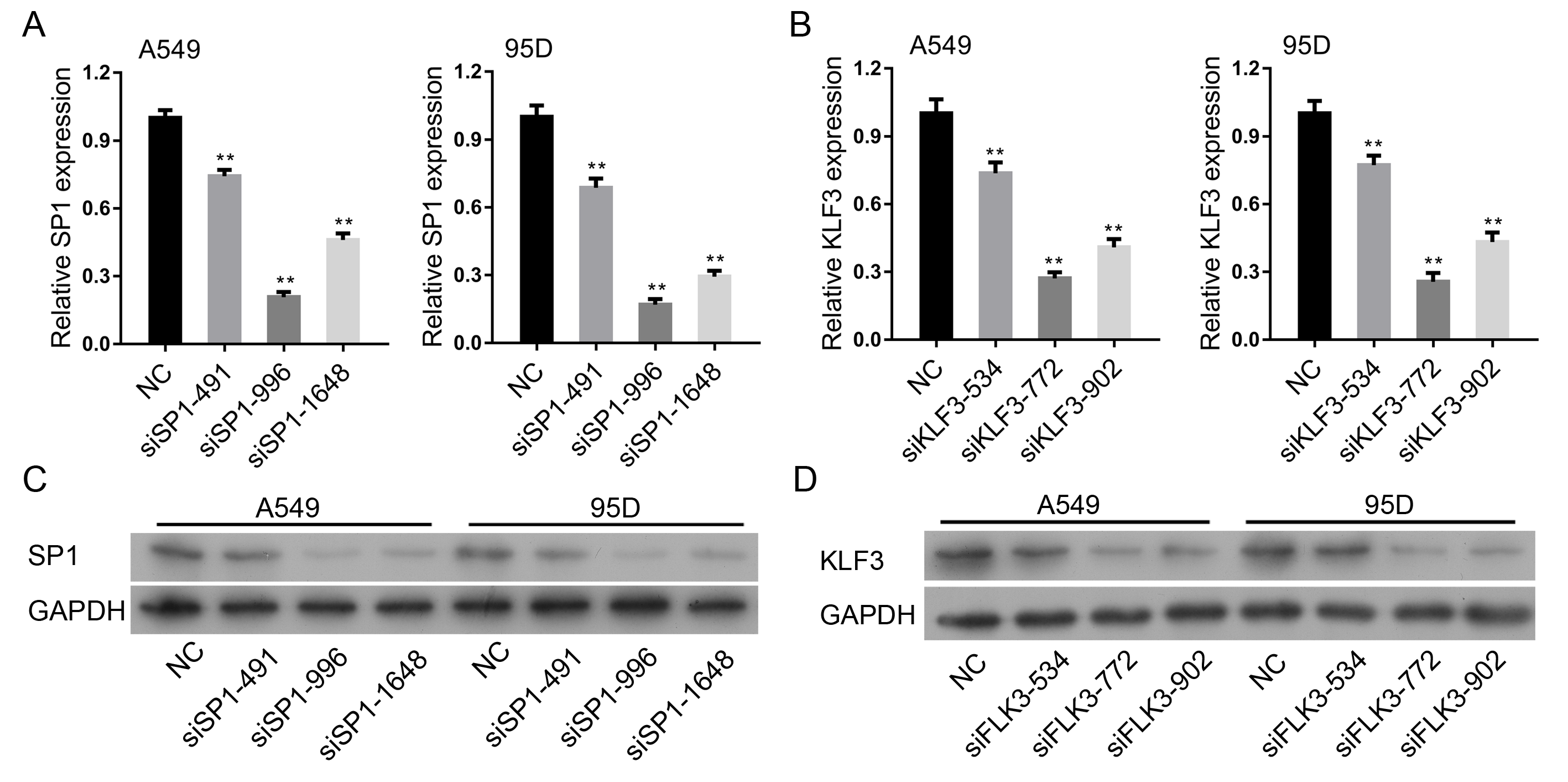

Supplement: Supplementary file 2 [file CPR-52-e12551-s002.tif]

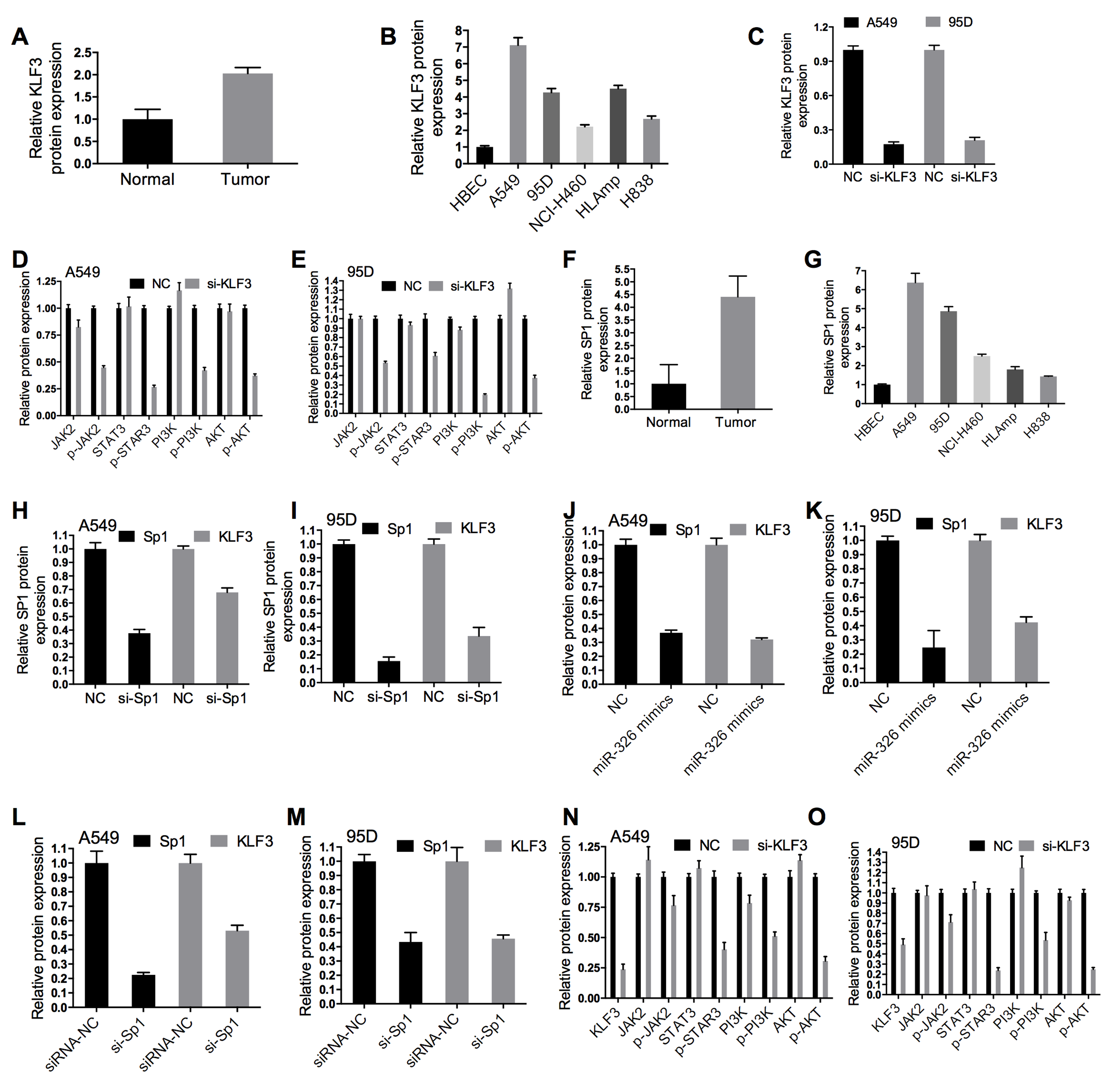

Supplement: Supplementary file 3 [file CPR-52-e12551-s003.tif]
