# Supplementary material for: MiR‐326/Sp1/KLF3: A novel regulatory axis in lung cancer progression
Source: Cell Prolif. 2018 Nov 28;52(2):e12551. doi: 10.1111/cpr.12551 (PMC6495967; doi:10.1111/cpr.12551)
Supplement: Supplementary file 4 [file CPR-52-e12551-s004.docx]

**Materials and methods**

**Cell culture**

Immortalized human bronchial epithelial cell line (HBEC), HBE1, was maintained in MEM medium supplement with 10% fetal bovine serum (FBS) and 1% penicillin/streptomycin. The lung cancer cell lines A549, 95D, NCI-H460, HLAmp and H838 were obtained from the American Type Culture Collection (ATCC) and cultured in RPMI-1640 supplement with 10% FBS (GIBCO) and 1% penicillin/streptomycin. All the cells were cultured under 37°C, 5% CO_2_.

**Clinical specimens**

Forty patients with lung cancer from January 2013 to December 2014 were enrolled in the First Affiliated Hospital of Nanjing Medical University, Jiangsu, China. Tumor and adjacent normal tissues were freshly collected during the surgery. All patients were pathologically and clinically diagnosed as Non-Small Cell lung cancer, NSCLC (Table 1). Patients’ consent and approval from the Ethics Committee of the First Affiliated Hospital of Nanjing Medical University (No: 2015-SRFA-021) were obtained before using these clinical materials for research purposes. Basic data regarding age, gender, Follow-up Duration (by December 2017), TNM (n, %) were collected.

**Real-time PCR and Western blot**

The tissue was grinded in liquid nitrogen and treated with Trizol (100 mg tissue: 1 ml Trizol). Then the solution was moved to an Ep tube and added with 200 μl chloroform. After vibrated for 15 s, the upper aqueous phase was added with 500 μl isopropanol for 10 min. After centrifuged at 12000 g for 10 min, the precipitation was added with 1 ml ethanol (75%). After centrifuged at 4 °C and 7500 g for 5 min, the supernatant was removed and the tube was dried for 10 min. RNA content and purity were determined by ultraviolet spectrophotometer. Reverse transcription was performed in a 20 μL with a system including 4μl 25mM MgCl_2_, 2μl Reverse Transcription 10X Buffer, 2μl 10mM dNTP Mixture, 0.5μl Ribonuclease Inhibitor, 15u AMV Reverse Transcriptase, 0.5μg Random Primers, 1μ total RNA and Nuclease-Free Water to a final volume of 20μl under 42 °C for 15 min and 85 °C denature (Promega, USA). Real-time PCR was then performed by using SYBR Premix Ex Taq GC kit (Takara, Japan) (7.5μl 2×premix, 10mM forword and reverse primers, dH_2_O to a final volume of 15μl) in the following condition: 94 °C denature for 30 sec, followed by 40 cycles each containing 94 °C denature for 5 sec, and 60 °C annealing for 30 sec with ABI 7500 (ABI, USA). Primer sequences were shown in Table 2. GAPDH was selected as internal reference. Relative gene expression was semiquantitative analyzed by 2^-△△Ct^ method. 2^-△△C^t = gene copy number in test group/gene copy number in control. Experiments were carried out in triplicates.

The whole cell lysate was prepared in RIPA buffer containing an anti-protease mixture (Roche, Mannheim, Germany). Proteins were resolved on SDS-PAGE gel, transferred into PVDF membranes, blocked with 5% nonfat milk for 1h, washed with PTBS for 3 times, and incubated with primary antibody (rabbit polyclonal anti-KLF3 antibody (Abcam, ab154531, Cambridge, USA), Anti-Sp1 antibody (Abcam, ab13370, Cambridge, USA), anti-JAK2 antibody (Abcam, ab205223, Cambridge, USA), anti-phospho-JAK2 antibody (Abcam, ab32101, Cambridge, USA), anti-STAT3 antibody (Abcam, ab119352, Cambridge, USA), anti-phospho-STAT3 antibody (Cell Signaling Technology, #9145, Beverly, USA), anti-PI3K antibody (Abcam, ab86714, Cambridge, USA), anti-phospho-PI3K antibody (Abcam, ab182651, Cambridge, USA), anti-AKT antibody (Abcam, ab8805, Cambridge, USA), anti-phospho-AKT antibody (Cell Signaling Technology, #4060, Beverly, USA), and anti-GAPDH (Cat#: KC-5G4, Kangchen, Shanghai, China) overnight at 4 °C. The membrane was then washed with PBST for 30 min, followed with incubation with HRP-conjugated goat anti-rabbit secondary antibodies (CST, 1:3000, Beverly, USA). The band was visualized using an ECL chemiluminescence substrate (Amersham Biosciences, USA). Gel image system was used to analyzed the band density (Bio-rad, Hercules, USA).

**Small interfering RNAs**

siRNAs were used to knockdown the expression of KLF3 and Sp1 in lung cancer cells. All the siRNA duplexes were purchased from GenePharma (GenePharma, Shanghai, China). The siRNA duplexes were transduced into lung cancer cells using Lipofectamine 2000 at a final concentration of 50 nM.　Sequences were shown in Table 3.

**Cell proliferation, migration and invasion**

Cells were seeded in 96-well plates, and assayed at 24, 48 and 72 hours using CCK-8 kit according to the manufacturer’s instructions. Cell viability was assessed by measurement of absorbance at 450 nm using a microplate reader.

Wound healing assay was used to assess cell migration ability of lung cancer cells. Briefly, the migration status was assessed by measuring the movement of cells into a scraped area created by a 200 μl pipette tip. After scratched, cells were washed twice and cultured in media supplemented with 0.1% FBS to eliminate the effect of cell proliferation. The spread of wound closure was photographed at 0 and 48 hours under ×5 objective lens.

For cell invasion assay, 5×10^4^ cells in serum-free media were placed in the top chamber (354480, BD Biosciences, San Jose, USA), while complete media was added to the bottom chamber. After incubated 24 hours at 37°C, the medium were removed and the chambers were fixed with methanol for 30 min, then were stained with crystal violet for another 30 min.

**Cell apoptosis and cell cycle**

Cell apoptosis were detected by Annexin V-FITC/PI double staining. Cells were harvested and washed with phosphate buffered solution (PBS), then incubated with 5 μl of an FITC-conjugated Annexin V and 5 μl PI for 10 min at room temperature in the dark. After incubation, the samples were analyzed by flow cytometry.

For cell cycle assay, cells were harvested and fixed in 70% ethanol for 48 h. The nuclei were stained with 50 μg/ml PI in 1% Triton-X100 containing 100μg/ml DNase-free RNase, and then analyzed by flow cytometry.

**Dual-luciferase reporter assay**

The KLF3 promoter region -1053/+183 construct was amplified from genomic DNA. The WT and mutated KLF3 promoter constructs were cloned into pGL3-Basic and verified by sequencing. Then, HEK293T cells were transfected with Sp1 expression plasmid and KLF3 promoter constructs by Lipofectamine 2000, followed by dual luciferase reporter assays (Promega, Madison, WI, USA). The results were expressed as ratio of firefly luciferase activity to Renilla. Similarly, human Sp1 3’UTR containing putative miR-326 binding sites were amplified by PCR from human genomic DNA of A549 cells. MiR-326 mimics or siRNA control with Sp1-3’UTR were co-transfected into293T cells respectively. After culturing 48 hours, the luciferase activity was analyzed using the Dual-Luciferase reporter assay system. Primer sequences were shown in Table 4 and differences of sequences between wild type and mutant were in Supplemental figure 1.

**Electrophoretic mobility shift assay (EMSA)**

EMSA was performed as described [1] by using the Light Shift chemiluminescent EMSA kit (Pierce, Rockford, IL, USA) according to the manufacturer’s instructions. Biotin-labeled double-stranded oligonucleotide containing the consensus Sp1 motif was used as EMSA probes. Unlabeled double-stranded oligonucleotide was used as competitor probe and the mutated oligonucleotide was used as negative control. Nuclear protein was extracted from A549 cells, and the antibody against Sp1 was used to supershift the DNA-protein complex.

**Chromatin Immunoprecipitation (ChIP)**

ChIP was performed by ChIP assay kit (17-371, EZ-CHIP, Millipore) according to the manufacture’s instruction. Briefly, lung cancer cells were fixed, lysed and sonicated to chromatins with an average size of 500 bp using a BioRuptor. Then, the chromatins were incubated with the anti-Sp1 antibody or rabbit IgG at 4°C with rotation overnight. The immunoprecipitated DNAs were purified by treatment with RNase A and proteinase K as well as cell extract DNAs (Input). Purified DNA was evaluated and analyzed by PCR with specific primers. The primers used for ChIP are as follows: KLF3-F: 5’-AGAGCGCGCTACAGGG-3’; KLF3-R: 5’-GCCAGGGCCATCGATCA-3’.

**Animal experiments**

The animal experiments were approved by the Ethics Committee of the First Affiliated Hospital of Nanjing Medical University, Jiangsu, China. BALB/c nude mice (age 4 weeks) were purchased from Laboratory Animal Center of Chinese Academy of Sciences (Shanghai, China). Briefly, NC siRNA or KLF3 siRNA infected A549 and 95D cell suspensions (0.1 ml, 5×10^5^ per mouse) were injected hypodermically into BALB/c nude mice (n = 5 per group). The tumor diameter in the width and length was measured using a verniercalliper every 3-4 days for a 4 weeks period. The tumor volume was calculated with the following formula: tumor volume (mm^3^) = 0.5×width (mm)^2^ × length (mm). Finally, all mice were put to death and the tumors were removed, weighed, and photographed.

**Immunohistochemical analysis**

The tumor tissues were dealt with 4% paraformald ehyde fixation, paraffin imbedding, cutting into slices to perform immunohistochemical analysis. Each tumor specimen was handled and stained according to the standard procedures using Histostain™-Plus 3rd Gen IHC Detection Kit (Invitrogen, Cat#: 85-9073, Carlsbad, USA) to examine the specific protein expression in human lung cancer tissues in nude mice. The antibodies used in immunohistochemical analysis were same as those in Western blot.

**Statistical analysis**

Data are presented as mean ± standard deviation of no smaller than three biological repeats. *t* test was used for the intergroup comparison, chi-square test was used for the enumeration data, and analysis of variance with Tukey’s post hoc test was used for the comparison among groups. Values of *p*<0.05 were considered statistically significant. All Statistical analysis was performed using the SPSS software (Version 11.6).

**Reference**

1. Lin Y, Yang Y, Li W, Chen Q, Li J, Pan X, Zhou L, Liu C, Chen C, He J *et al*: **Reciprocal regulation of Akt and Oct4 promotes the self-renewal and survival of embryonal carcinoma cells**. *Molecular cell* 2012, **48**(4):627-640.
